# Genome Sequence of Borrelia chilensis VA1, a South American Member of the Lyme Borreliosis Group

**DOI:** 10.1128/genomeA.01535-14

**Published:** 2015-02-12

**Authors:** Weihua Huang, Caroline Ojaimi, John T. Fallon, Dante Travisany, Alejandro Maass, Larisa Ivanova, Alexandra Tomova, Daniel González-Acuña, Henry P. Godfrey, Felipe C. Cabello

**Affiliations:** a Department of Pathology, New York Medical College, Valhalla, New York, USA; b Department of Microbiology and Immunology, New York Medical College, Valhalla, New York, USA; c Center of Mathematical Modeling and Center for Genomic Regulation, Universidad de Chile, Santiago, Chile; d Faculty of Medicine, Institute of Physiology, Comenius University, Bratislava, Slovakia; e Faculty of Veterinary Sciences, Universidad de Concepción, Chillán, Chile

## Abstract

Borrelia chilensis strain VA1 is a recently described South American member of the Borrelia burgdorferi *sensu lato* complex from Chile. Whole-genome sequencing analysis determined its linear chromosome and plasmids lp54 and cp26, confirmed its membership in the Lyme borreliosis group, and will open new research avenues regarding its pathogenic potential.

## GENOME ANNOUNCEMENT

The Borrelia genus contains three major groups of species (1, 2): the Lyme borreliosis group, several members of which cause Lyme disease throughout the Northern Hemisphere; the relapsing fever group, the members of which cause relapsing fever worldwide; and the reptile-associated group, the members of which infect reptiles but are not known to cause disease in humans. We recently identified and cultured a new borrelial species from Chile belonging to the Lyme borreliosis group (2). This new species, Borrelia chilensis VA1, isolated from Ixodes stilesi ticks present on environmental vegetation and long-tailed rice rats, has extended the range of the Lyme borreliosis group Borrelia to South America and the Southern Hemisphere.

We took advantage of high-throughput next-generation sequencing for whole-genome analysis, despite our inability to grow B. chilensis VA1 free of contaminating Delftia species (2). Genomic DNA was isolated from cultured spirochetes using the Qiagen (Valencia, CA) DNeasy blood and tissue kit. Two libraries were separately generated using the Illumina (Hayward, CA) Nextera XT DNA preparation kit and sequenced using a 251-bp paired-end library on the Illumina MiSeq system. The outputs were combined for a total of 13 million paired-end reads, reaching an estimated average coverage of about 50-fold. The sequences were assembled *de novo* using the Velvet algorithm with optimized *k*-mers (3). The resulting high-quality assemblies were mapped to Borrelia burgdorferi B31 (4) and Borrelia garinii BgVir (5) as reference genomes, and contaminating Delftia genomic DNA sequences were removed.

We obtained two complete contigs, one for linear plasmid lp54, and the other for circular plasmid cp26. We also identified 11 contigs for a scaffold of the chromosome. Short gaps in this scaffold were later closed by *in silico* analysis of mapping reads and contigs to genome references. The sequence redundancies between the *de novo* and mapped-to-reference assemblies were identical. The sequence annotations for both the chromosome and plasmids were performed using both Prokka 1.8 (6) and the NCBI Prokaryotic Genome Annotation Pipeline (7). No clustered regularly interspaced short palindromic repeats (CRISPR) were detected.

The linear chromosome contains 900,694 bp (G+C content, 28.5%), with 812 coding sequences (CDSs), 32 tRNAs, and five rRNAs. The linear plasmid lp54 contains 54,418 bp, with 64 CDSs, and circular plasmid cp26 contains 27,126 bp, with 27 CDSs. Pangenomic analysis using PGAP-1.11 (8) revealed that components on the chromosome and two plasmids of B. chilensis VA1 are syntenic with those of B. burgdorferi B31 and B. garinii BgVir. A whole-genome comparison with other borrelial species further confirmed B. chilensis VA1 as a new genospecies in the Lyme borreliosis group. Additionally, we identified a unique gene, *ndoR*, encoding a naphthalene 1,2-dioxygenase system ferredoxin-NAD^+^ reductase component, the role of which is uncharacterized in B. chilensis. Other plasmids containing more repetitive sequences remain to be assembled. The pathogenic potential of this new borrelial species for rodents and humans remains undetermined.

### Nucleotide sequence accession numbers.

The complete chromosome sequence of B. chilensis VA1 and the sequences of lp54 and cp26 have been deposited in the GenBank database under the accession numbers CP009910 to CP009912. This is the first version of the genome sequences for B. chilensis VA1.
